# High-Level Expression, Single-Step Immunoaffinity Purification and Characterization of Human Tetraspanin Membrane Protein CD81

**DOI:** 10.1371/journal.pone.0002314

**Published:** 2008-06-04

**Authors:** Hidehito Takayama, Prashen Chelikani, Philip J. Reeves, Shuguang Zhang, H. Gobind Khorana

**Affiliations:** 1 Center for Biomedical Engineering, Massachusetts Institute of Technology, Cambridge, Massachusetts, United States of America; 2 Mitsubishi Chemical Corporation, Yokohama, Japan; 3 Department of Biology, Massachusetts Institute of Technology, Cambridge, Massachusetts, United States of America; 4 Department of Chemistry, Massachusetts Institute of Technology, Cambridge, Massachusetts, United States of America; 5 Department of Biological Sciences, University of Essex, Colchester, United Kingdom; University of Arkansas, United States of America

## Abstract

The study of membrane protein structure and function requires their high-level expression and purification in fully functional form. We previously used a tetracycline-inducible stable mammalian cell line, HEK293S-TetR, for regulated high-level expression of G-protein coupled receptors. We here report successfully using this method for high-level expression of *de novo* oligo-DNA assembled human CD81 gene. CD81 is a member of the vital tetraspanin membrane protein family. It has recently been identified as the putative receptor for the Hepatitis C Virus envelope E2 glycoprotein (HCV-E2). In this study we used a single-step rho-1D4-affinity purification method to obtain >95% purity from HEK293S-TetR-inducible stable cell lines. Using ELISA assay we determined that the affinity of the purified CD81 receptor for HCV-E2 protein is 3.8±1.2 nM. Using fluorescent confocal microscopy we showed that the inducibly overexpressed CD81 receptor in HEK293S-TetR cells is correctly located on the plasma membrane. We demonstrated that the combination of high-level expression of CD81 with efficient single-step immunoaffinity purification is a useful method for obtaining large quantities of CD81 membrane receptor suitable for detailed structural analyses of this elusive tetraspanin protein. Furthermore, this simple single-step immunoaffinity purification to high purity of membrane protein could be useful broadly for other membrane protein purifications, thus accelerating the determination of structures for large numbers of difficult-to-obtain membrane proteins.

## Introduction

Recently enormous advancement has been made in high-resolution protein structural determinations, there are more than 50,000 protein structures and protein-complexes currently available in the Protein Data Bank (http://www.rcsb.org/pdb/home/home.do). However, except a few hundred minorities, almost of all the structures are soluble proteins.

It is known that membrane proteins are vital family proteins in all living systems. This is evident that ∼30% genes in almost all sequenced genomes code for membrane proteins [1]–[3]. However, our understanding of structures and functions of the membrane proteins lag far behind the known soluble proteins. As April 2008, there are only 157 unique membrane protein structures known among their variations of 368 total membrane proteins [http://blanco.biomol.uci.edu/Membrane_Proteins_xtal.html]. One of the reasons of lacking membrane protein structures is largely due to the notoriously difficult steps to purify large quantity of stable and functional membrane proteins. In order to accelerate membrane protein structural studies, new and simple methods are crucial.

**Figure 1:**
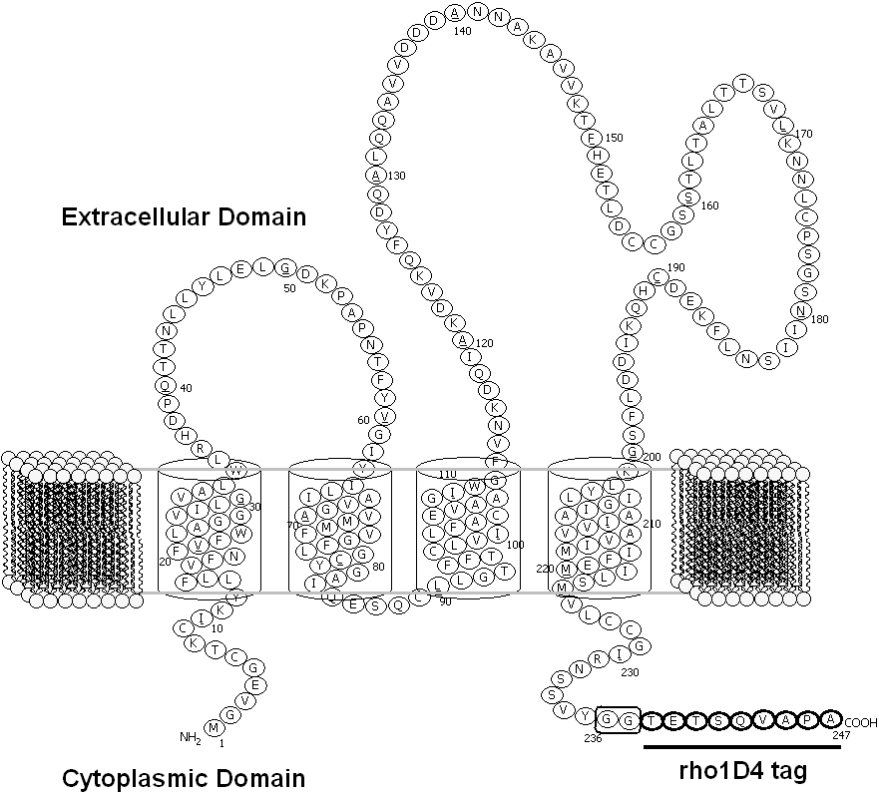
Schematic illustration of a two-dimensional model of human CD81 membrane receptor. The extracellular domain contains two loops, the small extracellular loop (SEL) and large extracellular loop (LEL). The CD81 receptor contains the rhodopsin C-terminal 9-residue peptide sequence (shown underlined) at its C-terminus as the tag to facilitate detection and purification of the membrane protein using the monoclonal antibody rho-1D4.

A very interesting and important class of integral membrane proteins is tetraspanins, which is a diverse family that comprises four transmembrane (TM) helices [4], [5]. Biochemical and bioinformatics analyses suggest that TM helix 1 is linked to TM helix 2 by a small extracellular loop (SEL). TM helix 3 is linked to TM helix 4 by a rather large extracellular loop (LEL) while an intracellular loop connects TM helices 2 and 3 [6], [7]. The LEL loop which contains four to eight cysteines and a signature Cys-Cys-Gly motif distinguishes the tetraspanins (Fig. 1) from other membrane proteins having four TMs. Although tetraspanins are widely distributed, occurring in most animal tissues and have been shown to be involved in essential cellular and physiological functions including cell proliferation, activation and signaling [4], [5], [7], its detail molecular structure remains largely elusive.

Human CD81 that belongs to a member of the tetraspanin family has been identified as the putative receptor for the Hepatitis C Virus envelope E2 glycoprotein (HCV-E2) [8]–[10]. Hepatitis C Virus is one of the leading causes of chronic liver diseases. According to a World Health Organization report, ∼3% of the human population is infected by HCV (WHO, 2002). Previously it has been shown that a recombinant soluble portion of CD81, designated CD81-LEL, binds to HCV-E2 with an affinity of 1.8 nM [8]. However, structural information on members of the tetraspanin family is rather limited. Of the relevant two studies, one reported on a high-resolution crystal structure of the CD81-LEL soluble domain fragment at 1.6Å [11], while a Cryo-Electron Microscopy (cryo-EM) study described a 6Å resolution structure of the entire tetraspanin, uroplakin [6]. The cryo-EM study of uroplakin suggests that both the transmembrane and extracellular domains of uroplakins are involved in interaction with other transmembrane proteins of the tetraspanin network.

Lack of a high-resolution structure for an entire tetraspanin is due to the lack of an expression system for preparation of the integral membrane protein in large quantity required for structural studies using protein X-ray crystallography or NMR spectroscopy. We previously reported the use of a tetracycline-inducible stable mammalian cell line (HEK293S-TetR) system for high-level expression of two G-protein coupled receptors (GPCR) of rhodopsin and β_2_-adrenergic receptor [12], [13]. We reasoned that we could use the same system to obtain large quantity of the elusive tetraspanin CD81. Here we report the successful use of this overexpression system with mammalian cell line for the high-level expression of CD81. We made a codon-optimized oligo-DNA/PCR synthesized gene, constructed a stable HEK293S-TetR cell line and inducibly expressed the gene. After high-level expression, we finally purified the CD81 protein using a single-step rho-1D4-affinity to near homogeneity. The yield of CD81 as visualized on immunoblots was ∼26 µg/3×10^7^ cells per 15 cm tissue culture plate. By using an ELISA assay, the affinity of the purified CD81 receptor for HCV-E2 protein was estimated to be 3.8±1.2 nM. This simple assay, which involves in coating of the rho-1D4-antibody on ELISA plates followed by immobilization of CD81, will have applications for high-throughput screening (HTS) of libraries of small molecule inhibitors of CD81. Biochemical analysis of the purified CD81 showed no detectable post-translational modifications including N- and O-glycosylations that are normally found in other tetraspanin membrane proteins expressed in mammalian cells.

## Results and Discussion

### Designed gene and mammalian overexpression

The codon optimized synthetic DNA corresponding to the human CD81 gene comprises 776 base-pairs of which 744 base-pair represents the open reading frame (Fig. S1). The design and synthesis of this gene is described in the Methods section. To ensure tightly regulated high-level expression of this potentially toxic CD81 gene, stable cell lines of CD81 were constructed using the HEK293S-TetR inducible system. A total of 32 independent clones were examined for the expression of CD81 using a dot-blot immuno-detection method. The cell line that showed the highest protein expression was selected for further studies. HEK293S-TetR cells expressing the synthetic CD81 gene were grown as monolayers in 15 cm tissue culture dishes. After induction with tetracycline and sodium butyrate for ∼44–48 hours, the cells were collected by centrifugation and the cell pellet was quick-frozen and stored at −80°C until purification. After induction, a relatively high binding of anti-CD81 antibody on the cell surface of HEK293S-TetR detected using cellular ELISA assay (data not shown) indicated the presence of overexpressed CD81 on the plasma membrane.

### Single-step immuno-affinity purification of CD81 membrane protein

**Figure 2:**
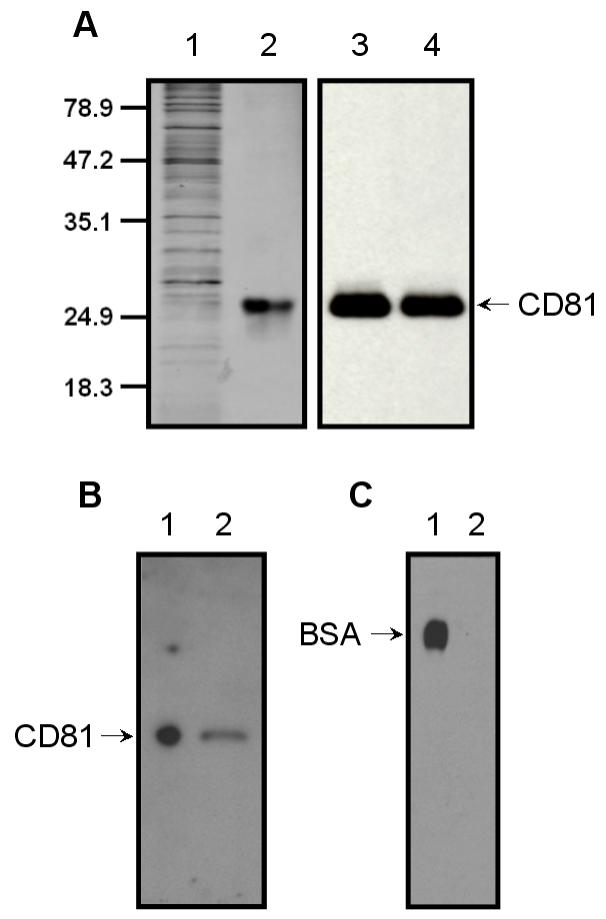
Affinity purification of CD81 receptor. A) SDS-PAGE (16%) of the solubilized (Lane 1) and rho-1D4-affinity purified CD81 (Lane 2). Proteins were detected by silver staining. Lane 1, 2.5 µg of solubilized membrane protein, Lane 2, 0.5 µg of rho-1D4-purified receptor. Lanes 3 and 4 represent the immunoblot analysis of the same samples as in lanes 1 and 2, but same volume fraction of each solubilized and purified sample (1/7000) was loaded. Immunoblot analysis was done using the monoclonal antibody rho-1D4 (the epitope tag for this antibody was added to the C-terminal tail of CD81 gene). Mobility of molecular weight standards is indicated next to the gel. B) Immunoblot analysis of PNGaseF treated (Lane 1) and an untreated (Lane 2) sample of CD81, 1 µg of total solubilized protein is loaded in each lane. C) Immunoblot analysis of CD81, with a highly specific mouse monoclonal antibody for the detection of *O*-GlcNAc post-translational modification. As a positive control, *O*-β*-*GlcNAc-modified BSA (5 ng), which runs as a 66kDa band, is loaded in lane 1. In lane 2, 1 µg of total solubilized CD81 protein is loaded.

For receptor purification, cell membranes were prepared from the cell pellet and solubilized using buffer B as described in Materials and Methods. The receptor purified using the rho-1D4-Sepharose beads was >95% purity as determined by SDS-PAGE and silver staining (Fig. 2A). The yield of CD81 following the single-step rho-1D4-Sepharose affinity purification was determined by semi-quantitative immuno-detection using the signal generated by known amounts of rhodopsin (isolated from bovine retina) for calibration (Fig. S2). This quantitative method of protein concentration showed the expression of CD81 to be ∼26 µg/3×10^7^cells per 15 cm tissue culture plate. While this level of expression is one of the highest reported so far for the total CD81 receptor using mammalian cell, previous studies on the expression of soluble CD81-LEL in *Escherichia coli* using a GST-LEL fusion tag reported expression level of up to 3 mg/L, and achieved a purification of >95% using four step purification procedures [10].

### Characterization of purified CD81 tetraspanin membrane protein

**Figure 3:**
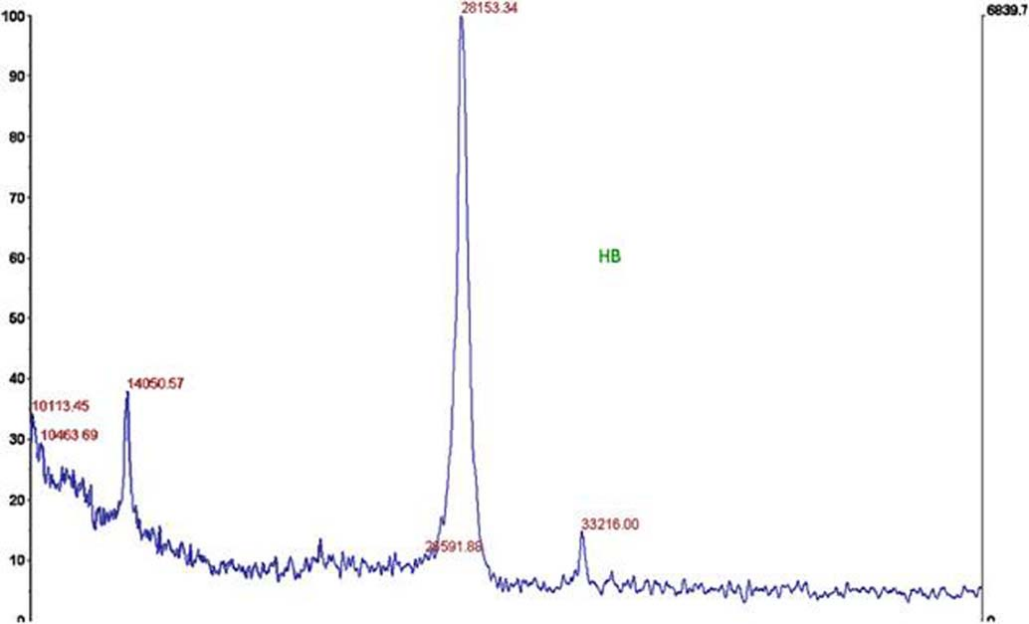
MALDI-TOF mass analysis of anti-rho-tag affinity-purified CD81 receptor membrane protein. The peak at 28,153 daltons corresponds to the purified CD81. The sample is internally calibrated using a standard, which is observed as a peak at 33,216 daltons.

The purified CD81 migrated as a single band at around 27kDa on 16% SDS-PAGE (Fig. 2A). To separate the nonapeptide from the eluted pure protein, the purified CD81 was concentrated using a 10-kDa cut off membrane (Amicon YM-10). However, MALDI-TOF mass analysis of the purified protein after desalting showed a mass of 28,153 Daltons (Fig 3), indicating a discrepancy of 1,345 Daltons between the observed and the theoretical mass, 26,808 Daltons. The extra mass could be due to the presence of the non-ionic detergent remaining bound or interacting to the protein or might be due to other post-translational modifications. The N-terminus of the purified protein was confirmed by N-terminal sequencing to have the amino acid sequence M/G, G/V, V/E, E/G, and G and since the C-terminus of the protein carried the rho-1D4 affinity tag, this confirmed the purified CD81 to be the full length protein. Further, the observed discrepancy in mass is not uncommon among the tetraspanins, with a recent report showing the theoretical mass of CD9 to be 25 kDa while the observed mass was found to be 27kDa [14].

The extracellular sequence of CD81 lacks the consensus N-glycosylation motif, Asn-xxx-Ser/Thr, thus as expected, the receptor expressed in the HEK293S-TetR stable cell line did not appear N-glycosylated because its migration on SDS-PAGE was unaltered even after treatment with N-glycanase PNGaseF (Fig 2B). This result is in agreement with previous observation that CD81, unlike other tetraspanins, is unglycosylated [15]. The possibility that CD81 had other post-translational modification, such as O-glycosylation (*O*-GlcNAc), was investigated. Immuno-blottting of CD81 followed by probing of the blot with the highly specific mouse monoclonal antibody for the detection of the *O*-GlcNAc post-translational modification (see Methods) showed that CD81 did not have any *O*-GlcNAc (Fig 2C).

### Sub-cellular localization of CD81 in HEK293S cells

**Figure 4:**
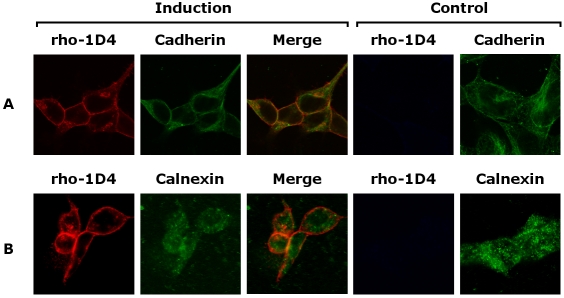
Localization of CD81 overexpressed in HEK293S-TetR cell line. Following induction of cells harboring HEK293S-TetR-CD81, the cells were fixed with formaldehyde, and permeabilized with 0.05% Triton X-100. Dual immuno-fluorescence was performed with mouse rho-1D4 antibody and rabbit anti-cadherin antibody (A) or anti-calnexin antibody (B), which were localized at the plasma membrane and ER, respectively. Mouse rho-1D4 antibody was visualized with Alexa 647 anti-mouse secondary antibody (red), and rabbit anti-cadherin and calnexin antibody was visualized with Alexa 488 anti-rabbit secondary antibody (green). Overexpressed CD81-rho-1D4 was localized at the plasma membrane (merge), while endogenous CD81-rho-1D4 was not observed without induction (control).

Since we made the synthetic CD81 membrane protein gene, we are interested if CD81 is functional and indeed localizes on the cell plasma membrane. We therefore carried out experiments to determine the sub-cellular localization of CD81 expressed in the inducible HEK293S stable cell lines using confocal fluorescent microscopy. We examined the sub-cellular distribution of CD81 in the HEK293S-TetR cells, before and after induction with tetracycline and sodium butyrate, using rho-1D4 antibody for immunofluorescence staining (see Methods). We found that the overexpressed CD81 was mainly localized at the cell surface (Fig. 4) with only minimal amounts, presumably in-transit, present intracellularly. This localization result is a strong indication of correct folding of CD81 because misfolded membrane proteins are retained in the ER by the highly effective ER quality control system [16]. Owing to the tight repression by tetR protein, in the absence of the inducers, tetracycline and sodium butyrate, HEK293S-TetR cells harboring the CD81 gene did not show detectable fluorescent signal from leaky expression of CD81 gene.

### Affinity of purified CD81 for HCV-E2 binding assay

**Figure 5:**
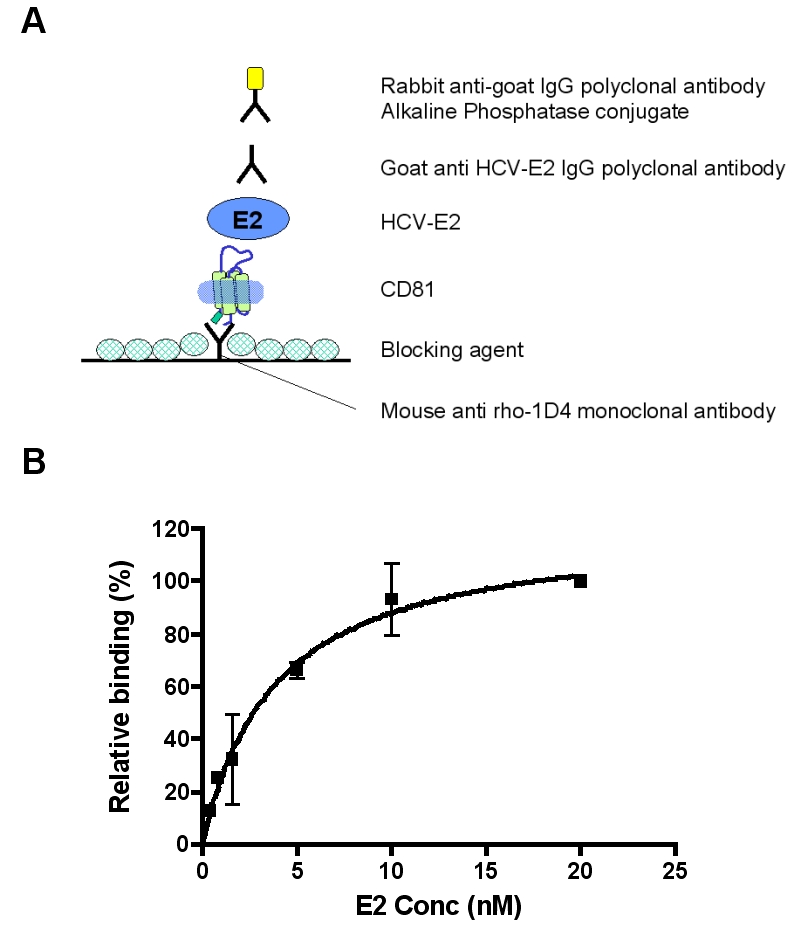
Binding affinity of purified CD81 for HCV-E2. A) Schematic showing the methodology of the ELISA assays using rho-1D4 antibody and purified CD81 receptor. B) ELISA plates were coated with rho-1D4 monoclonal antibody and purified CD81 was added, followed by the addition of increasing concentrations of HCV-E2 from 0.4 to 20 nM (assay was carried out as described in Materials and Methods). Specific binding expressed as percentage, was derived by subtracting nonspecific binding (binding in the absence of rho-1D4) from the total binding. Binding data were obtained from a minimum of two independent determinations done in duplicate and are shown as mean±S.E. Data were analyzed by non-linear regression using PRISM software (Graph Pad Software, San Diego, CA).

Measuring the binding affinity of CD81-LEL to HCV-E2 glycoprotein is an established method to characterize the functional integrity of CD81. While most of the preceding studies have used only the LEL-fragment of CD81 to characterize HCV-E2 binding, here we used the entire purified detergent DM-solubilized CD81 receptor and measured its affinity towards HCV-E2 in an *in vitro* assay. In this assay CD81 was oriented to favor binding to HCV-E2, accomplished by coating the plates with rho-1D4 antibody (Fig. 5A). The epitope tag for this antibody is present at the C-terminus of CD81 so that the extracellular region of CD81 is free for binding to HCV-E2. Using this assay, the affinity between the purified CD81 and HCV-E2 was found to be 3.8±1.2 nM (Fig. 5B), a value close to the previously reported affinity, 1.8 nM, between CD81-LEL and HCV-E2 [5]. The observed difference in affinity could be due to the fact that the entire CD81 was used as only a water-soluble fragment CD81-LEL, which could have fold differently and could have tendency of binding with more exposed conformation. Also, the presence of the detergent DM could influence the affinity of the interaction. However, it is plausible our measurement is closer to the cellular CD81 receptor binding affinity to HCV-E2 when measured both with the entire receptor protein and in the presence of membrane protein-stabilization detergent.

### Biomedical application of the affinity purified CD81

One of the possible applications of this study is to enable high-throughput screening (HTS) to rapidly identify inhibitors for CD81-HCV-E2 binding. A prerequisite for this step is the availability of obtaining large quantity of highly purified CD81, as we have now demonstrated. Furthermore, immobilization of CD81 receptor at the C-terminus using rho-1D4 antibody ensures that the E2 binding site (CD81-LEL) remains fully accessible. Several surface modification techniques such as polymer coating, site-directed immobilization and gel network are available to maximize the binding capacity of surface-coating protein (rho-1D4 antibody in this study) and to immobilize in favorable orientation, and prevent denaturation during immobilization [17].

A well-structured 3D surface rather than a 2D surface can also be microfabricated to significantly increase the surface area to sample volume ratio [18]. In addition, miniaturization of reaction space significantly decreases the sample volume which, in turn, reduces the reaction time to reach equilibrium [19]. These improvements utilizing multidisciplinary techniques such as, surface chemistry, microfluidics and microfabrication [19], combined with membrane protein biochemistry offer promise to improve sensitivity and reproducibility for HTS.

We have developed an efficient system for the functional expression with mammalian cell line and a single-step immunoaffinity purification of human CD81 tetraspanin membrane receptor. Using this system, purification of the receptor in milligram to gram quantities is now feasible using suspension culture in a large bioreactor. Production of CD81 at such high-levels will not only enable detailed structural analysis of this elusive family of integral tetraspanin membrane proteins, but also allow biotechnological advancement to immobilize CD81 on several formats of solid supports for drug discovery and disease diagnosis to combat HCV infectious liver diseases.

### A useful and simple method for obtain large quantity of membrane proteins

The combination of using rhodopsin affinity 9-residue motif to tag onto other membrane proteins and using anti-rho-tag antibody as the affinity purification method could have far reaching implications for quickly obtaining large quantity membrane proteins. We have also applied this simple method to purify large quantities of several *de novo* assembled human and mouse olfactory receptor proteins. The methods we reported here demonstrated that this simple single-step immunoaffinity purification to extremely high purity could be useful broadly for other membrane protein purifications, thus accelerate determination of the structures of large number of difficult-to-obtain membrane proteins.

## Materials and Methods

### Materials

Synthetic oligonucleotides were purchased from IDT (Coralville, IA). Sepharose 4B was purchased from Sigma (St.Louis, MO). The detergent *n*-dodecyl-β-D-maltoside (DM) was purchased from Anatrace (Maumee, OH). The monoclonal antibody, rho-1D4, was prepared by the Cell Culture Center (Minneapolis, MN) from a cell line provided by R.S. Molday (University of British Columbia, Vancouver, Canada). FBS and tetracycline were purchased from Sigma, and sodium butyrate was from J. T. Baker (Phillipsburg, NJ). Ham's F-12/DME High Glucose was from Irvine scientific (Santa Ana, CA). Geneticin (G418), Blasticidin S-HCl was from Invitrogen (Carlsbad, CA). Protease inhibitors and common chemicals were purchased either from Sigma or Invitrogen. Restriction enzymes and PNGase F were purchased from New England Biolabs (Ipswich, MA). The 9-residue peptide corresponding to the C-terminal sequence of rhodopsin, which was used to elute CD81 from the antibody rho-1D4 sepharose matrix, was synthesized at the MIT-Biopolymers Laboratory (Cambridge, MA).

Immulon ELISA plates were from Santa Cruz (Santa Cruz, CA). ELISA amplification system is from Invitrogen. Rabbit anti-cadherin and calnexin polyclonal antibody, Goat anti-human Hepatitis C Virus E2 polyclonal antibody and Rabbit anti-goat IgG with alkaline phosphatase conjugate was from Abcam (Cambridge, MA). Recombinant HCV E2 envelope protein was from Immuno Diagnostics (Woburn, MA). Ultrablock (blocking solution) was from Serotec (Raleigh, NC). BioCoat Collagen I 8- well culture slide was from BD Falcon (Franklin Lakes, NJ). Image-iT FX Signal enhancer, Alexa Fluor 488 goat anti-rabbit antibody and 647 goat anti-mouse antibody were from Invitrogen.

Buffers used were as follows: PBS buffer: 137 mM NaCl, 2.7 mM KCl, 1.8 mM KH_2_PO_4,_ 10 mM Na_2_HPO_4_ (pH 7.4); Buffer A (Lysis buffer), 10 mM Tris-HCl, pH 7.4, containing protease inhibitors (1 mM EDTA, 10 µg/ml benzamidine, 10 µg/ml leupeptin, 20 µg/ml soybean trypsin inhibitor, 5 µg/ml aprotinin, and 0.2 mM phenylmethylsulfonyl fluoride); Buffer B (Solubilization buffer), 20 mM Tris- HCl, pH7.4, containing 500 mM NaCl, 10% glycerol, 1% DM and the protease inhibitors as in buffer A; Buffer C (High-salt buffer), 20 mM Tris-HCl, pH7.4 containing 500 mM NaCl; Buffer D (TBS buffer), 25 mM Tris-HCl, pH 7.5 containing 144 mM NaCl.

### Methods

#### Synthesis, cloning and stable cell line construction of human CD81 gene

The synthetic CD81 gene consisted of 776bp, of which the 744bp encodes CD81-GlyGly-rho-1D4. The salient features of the codon optimized CD81 gene include, a Kozak consensus (**GCCACCATGG**) 5′ of the ATG start codon [20], a two amino acid long glycine spacer and the bovine rhodopsin C9 epitope tag (**TETSQVAPA**) immediately 5′ to the natural stop codon of CD81, and restriction sites for EcoRI at the 5′ end and NotI at the 3′ end to facilitate cloning into expression vectors. The sequence encoding the human CD81 gene (NCBI Accession # BC002978) was optimized for mammalian codon usage by utilizing the codons predicted to occur frequently in highly expressed genes of mammals [21]. PCR primers were designed using DNAWorks [22] http://mcl1.ncifcrf.gov/dnaworks/dnaworks2.html. A total of 40 oligonucleotides were synthesized at 25 nmol scale with the sense strand (referred as SS) and the antisense strand (referred as AS) consisting of 20 oligonucleotides each (Table S1). Construction of the human CD81 gene and its cloning into the pACMV-tetO vector [12] and the subsequent construction of HEK293S tetracycline inducible stable cell lines was similar to what has been reported before for beta_2_-adrenergic receptor [13], except the PCR program for gene synthesis consisted of 30 cycles.

#### Localization of overexpressed CD81

Changes in CD81 cell localization after induction was measured using confocal microscopy. HEK293S-TetR-CD81 cells were cultured on BD BioCoat culture slides, 24 hours or 48 hours after induction the wells were washed with 1× PBS, and then fixed in 3.7% formaldehyde/1× PBS for 15 min at room temperature. The cells were then permeablized with 0.05% triton X-100/1× PBS for 30 min at room temperature. The permeablization buffer was then washed out with 1× PBS, the cells blocked with Signal Enhancer (Invitrogen), blocking solution, incubated with the primary antibody (mouse rho-1D4, rabbit anti-cadherin, rabbit anti-calnexin) in 10% FBS/1× PBS for 1 hour at room temperature, washed, incubated with a fluorescent coupled secondary antibody (Alexa Fluor™ 488 goat anti-rabbit , Alexa Fluor™ 647 goat anti-mouse), washed twice, then covered with coverglass and nail polish. The slides were visualized using Radiance 2100 MP laser confocal microscope and LaserSharp 2000 software (Bio-Rad). Images were processed using ImageJ (NIH, Bethesda, MD).

#### Purification of CD81 by rho-1D4 affinity chromatography

Tetracycline inducible HEK293S stable cell lines expressing CD81 were grown to confluence in 15 cm dishes and expression of CD81 was induced by adding 1 µg/ml tetracycline and 5 mM sodium butyrate. Following induction for 44 hours, the dishes were rinsed, using ice-cold PBS buffer and the cells were then harvested in buffer A. All further steps were carried out at 0° to +4°C. The cell suspension was next transferred into a 15 ml dounce tissue homogenizer and the cells were homogenized using 15 strokes. The cell homogenate was centrifuged at 48,000× g for 20 min at 4°C. The resulting membrane pellet was snap frozen in liquid nitrogen and stored at −80°C until required. The protein concentration in the resuspended membrane pellet was determined using DC protein assay kit from Bio-Rad Laboratories (Hercules, CA). Cell pellets from 10 to 40 dishes (15 cm), were resuspended using buffer B, and the suspension was further homogenized using a dounce homogenizer (15 strokes). The solubilized CD81 receptor was adsorbed to rho-1D4-Sepharose beads in batch mode (binding capacity corresponding to 1 mg rhodopsin/ml of beads) with slow agitation for 2–4 h at 4°C. The rho-1D4-beads were then collected by centrifugation at 1,500 g and washed with buffer C until the absorbance of the wash at 280 nm was below 0.01. Elution was carried out with buffer C containing 100 µM nonapeptide. The receptor appeared in the elution, following elution with 2-bed volumes of the eluant. Elution samples were concentrated by using Amicon YM-10 (Millipore, Bedford, MA). The protein concentration was determined by BioRad DC protein assay.

#### Quantitation of CD81 by Immunoblots

The total amount of CD81 receptor obtained by the single-step affinity purification was determined by using quantitative immunoblot analysis. For these assays, known amounts of purified bovine rhodopsin (200, 400, 600, 800 and 1000 pg) were used to generate a standard curve. CD81 purified as described above was diluted in PBS buffer containing 0.05% DM, to achieve a dilution within the range of the standards. Protein samples were resolved by 12% SDS-PAGE gel. The protein was then transferred from the gels onto a nitrocellulose membrane by electroblotting and CD81 along with bovine rhodopsin was visualized by immuno-detection with the monoclonal antibody, rho-1D4 followed by incubation with HRP-linked anti mouse IgG and ECL. The strength of chemiluminesence signal on the blots was quantified either by a phosphoimager or visualized by using Kodak X-OMAT film. The pixel density was determined using Scion Image for Windows (Scion Corporation, Frederick, MD). The data were fit using a linear regression algorithm in Sigma plot v8.0 (SPSS Science, Chicago, IL).

#### Glycosylation analysis

Cell membranes containing CD81 were solubilized with 1% DM and were treated with PNGaseF and incubated for 1 h at 37°C. Separation of reaction products are visualized by SDS-PAGE. The presence of O-linked glycosylation (*O*-GlcNAc) was determined by immunoblot analysis using the *O*-GlcNAc Western Blot Detection system from Pierce (Rockford, IL), and according to the directions supplied by the manufacture. As a positive control, *O*-GlcNAc-modified BSA, which runs at approximately 66 kDa, was used (Fig. 2C).

#### Mass spectrometry and N-terminal sequencing

The MALDI-TOF spectra analysis of the rho-1D4 purified CD81 sample was conducted by the MIT Biopolymers laboratory. Briefly, purified CD81 was concentrated using Amicon YM-10 to 0.25 mg/ml concentration and processed using a Zip-Tip. The sample was then reconstituted in 4 µl of 50% ACN/0.1% TFA. Then, 3 µl of the sample was dried down, reconstituted in 1 µl of matrix (sinapinic acid), and mass spectra were obtained in linear mode. The sample was internally calibrated using a standard, which was observed as a peak at 33,216 Da. N-terminal sequencing was performed by Toray Research Center, Inc, Tokyo, Japan.

#### ELISA assay for measuring the affinity of purified CD81 for HCV-E2

The ability of purified CD81 to bind to HCV-E2 was measured using ELISA. ELISA plates (96-well) were coated with 100 ng of rho-1D4 antibody in PBS buffer (200 µl). Following overnight incubation at 4°C, the unbound rho-1D4 antibody was removed by three washes, each with 200 µl TBS buffer. All further steps were carried out at room temperature. To minimize non-specific binding the wells were blocked with Ultra-block for 2 hours. This was followed by addition of 100 ng of purified CD81 (in buffer D containing 0.05% DM) to the wells, following incubation for another 2 h; various concentrations of E2 glycoprotein from 0.4 to 20 nM (in buffer D containing 0.05% DM) were now added to the wells and binding allowed to proceed for another 2 h. Primary anti-E2 antibody at 1∶2000 dilutions and secondary antibody with alkaline phosphatase conjugate at 1∶1000 dilutions were added followed by incubation for another 2 h. Enzymatic color reaction was carried out using ELISA amplification system following the manufacturers instructions. Measurements were recorded at the absorbance of 495 nm by Spectra MAX 340PC, Molecular Devices.

## Supporting Information

Figure S1 Nucleotide sequence of the codon-optimized CD81 gene, and the corresponding amino acid sequence. The locations of the restriction sites EcoRI and NotI in the gene are shown above the DNA sequence. The synthetic gene contains at its C-terminus the rhodopsin C-terminal nonapeptide sequence (shown underlined) to facilitate detection and purification of the protein using the monoclonal antibody rho-1D4.
(0.02 MB DOC)

Figure S2 Quantitative CD81 receptor expressed in HEK293S-TetR stable cell line. Left panel, Immunoblot analysis of CD81 using monoclonal antibody rho-1D4, the mobility of CD81 corresponded to a molecular mass of about 27kDa. The increasing amounts of rhodopsin standards (ROS) allow determination of the amount of CD81 expressed. Right panel, determination of CD81 expression by comparison to rhodopsin standards shown in the left panel. By this method, the amount of rho-1D4 affinity purified CD81 obtained was determined to be 26±2 µg/3×107 cells (15 cm tissue culture plate).
(0.23 MB TIF)

Table S1 PCR primers used for synthesis of the codon-optimized CD81 gene. The sense strand (SS) and anti-sense strand (AS) consist of 20 oligonucleotides each, and the sizes of the primers in base pairs are indicated next to the sequence.
(0.02 MB DOC)
