# Supplementary material for: High-Level Expression, Single-Step Immunoaffinity Purification and Characterization of Human Tetraspanin Membrane Protein CD81
Source: PLoS One. 2008 Jun 4;3(6):e2314. doi: 10.1371/journal.pone.0002314 (PMC2391292; doi:10.1371/journal.pone.0002314)
Supplement: Figure S1 — Nucleotide sequence of the codon-optimized CD81 gene, and the corresponding amino acid sequence. The locations of the restriction sites EcoRI and NotI in the gene are shown above the DNA sequence. The synthetic gene contains at its C-terminus the rhodopsin C-terminal nonapeptide sequence (shown underlined) to facilitate detection and purification of the protein using the monoclonal antibody rho-1D4. (0.02 MB DOC) [file pone.0002314.s001.doc]

**SUPPLEMENTARY INFORMATION**

**Figure S1.** Nucleotide sequence of the codon-optimized CD81 gene, and the corresponding amino acid sequence. The locations of the restriction sites EcoRI and NotI in the gene are shown above the DNA sequence. The synthetic gene contains at its C-terminus the rhodopsin C-terminal nonapeptide sequence (shown underlined) to facilitate detection and purification of the protein using the monoclonal antibody rho-1D4.

**EcoRI**

cctgaattcgccgccaccatgggcgtggagggctgcacaaagtgtattaagtacctgctg

M G V E G C T K C I K Y L L

ttcgtgttcaactttgtgttctggctcgccggaggcgtgattctcggagtggctctctgg

F V F N F V F W L A G G V I L G V A L W

ctcagacatgacccccagacaacaaatctgctgtacctggaactcggagacaagcccgct

L R H D P Q T T N L L Y L E L G D K P A

cctaatacattctatgtgggaatctacatcctgattgccgtcggcgccgtgatgatgttt

P N T F Y V G I Y I L I A V G A V M M F

gtcggattcctgggatgctacggagctattcaagagagccaatgtctcctcggaaccttc

V G F L G C Y G A I Q E S Q C L L G T F

tttacctgcctcgtcatcctgttcgcttgtgaagtcgctgccggaatttggggatttgtc

F T C L V I L F A C E V A A G I W G F V

aacaaagaccagattgctaaggacgtgaaacagttctacgaccaggccctgcaacaggct

N K D Q I A K D V K Q F Y D Q A L Q Q A

gtggtggatgacgatgccaacaacgctaaagctgtcgtgaagaccttccatgagacactg

V V D D D A N N A K A V V K T F H E T L

gattgttgtggaagcagcaccctgaccgccctgaccaccagcgtgctcaagaacaatctg

D C C G S S T L T A L T T S V L K N N L

tgtcctagcggatccaacatcatttccaacctgttcaaagaggattgccaccagaaaatt

C P S G S N I I S N L F K E D C H Q K I

gatgatctgtttagcggcaagctctacctcatcggcatcgccgccatcgtcgtggctgtc

D D L F S G K L Y L I G I A A I V V A V

atcatgattttcgagatgattctcagcatggtcctctgctgcggaattagaaactcctcc

I M I F E M I L S M V L C C G I R N S S

**NotI**

gtctacggcggaaccgagacctcccaagtggctcccgcttgagcggccgcgagaag

V Y G G T E T S Q V A P A *
