# Supplementary figures and images for: High-Level Expression, Single-Step Immunoaffinity Purification and Characterization of Human Tetraspanin Membrane Protein CD81
Source: PLoS One. 2008 Jun 4;3(6):e2314. doi: 10.1371/journal.pone.0002314 (PMC2391292; doi:10.1371/journal.pone.0002314)

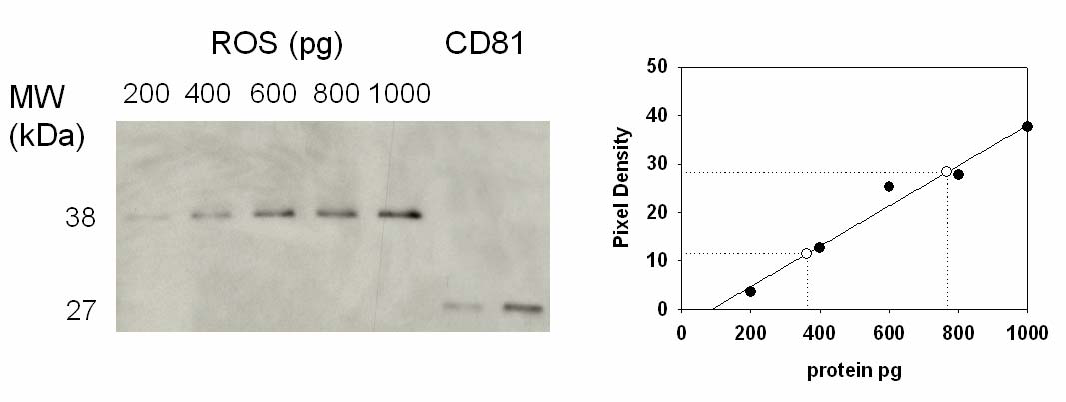

Supplement: Figure S2 — Quantitative CD81 receptor expressed in HEK293S-TetR stable cell line. Left panel, Immunoblot analysis of CD81 using monoclonal antibody rho-1D4, the mobility of CD81 corresponded to a molecular mass of about 27kDa. The increasing amounts of rhodopsin standards (ROS) allow determination of the amount of CD81 expressed. Right panel, determination of CD81 expression by comparison to rhodopsin standards shown in the left panel. By this method, the amount of rho-1D4 affinity purified CD81 obtained was determined to be 26±2 µg/3×107 cells (15 cm tissue culture plate). (0.23 MB TIF) [file pone.0002314.s002.tif]
