# Supplementary material for: High-Level Expression, Single-Step Immunoaffinity Purification and Characterization of Human Tetraspanin Membrane Protein CD81
Source: PLoS One. 2008 Jun 4;3(6):e2314. doi: 10.1371/journal.pone.0002314 (PMC2391292; doi:10.1371/journal.pone.0002314)
Supplement: Table S1 — PCR primers used for synthesis of the codon-optimized CD81 gene. The sense strand (SS) and anti-sense strand (AS) consist of 20 oligonucleotides each, and the sizes of the primers in base pairs are indicated next to the sequence. (0.02 MB DOC) [file pone.0002314.s003.doc]

**Table S1:** PCR primers used for synthesis of the codon-optimized CD81 gene. The sense strand (SS) and anti-sense strand (AS) consist of 20 oligonucleotides each, and the sizes of the primers in base pairs are indicated next to the sequence.

*SS1. CCTGAATTCGCCGCCACCATGGGCGTGGAGG 31bp

SS2. GCTGCACAAAGTGTATTAAGTACCTGCTGTTCGTGTTCAACTTTG 45bp

SS3. TGTTCTGGCTCGCCGGAGGCGTGATTCTCGGAG 33bp

SS4. TGGCTCTCTGGCTCAGACATGACCCCCAGACAACAAA 37bp

SS5. TCTGCTGTACCTGGAACTCGGAGACAAGCCCGCT 34bp

SS6. CCTAATACATTCTATGTGGGAATCTACATCCTGATTGCCGTCGGC 45bp

SS7. GCCGTGATGATGTTTGTCGGATTCCTGGGATGCTACGG 38bp

SS8. AGCTATTCAAGAGAGCCAATGTCTCCTCGGAACCTTCTTTACCT 44bp

SS9. GCCTCGTCATCCTGTTCGCTTGTGAAGTCGCTGC 34bp

SS10. CGGAATTTGGGGATTTGTCAACAAAGACCAGATTGCTAAGGACG 44bp

SS11. TGAAACAGTTCTACGACCAGGCCCTGCAACAGGCTGT 37bp

SS12. GGTGGATGACGATGCCAACAACGCTAAAGCTGTCGTG 37bp

SS13. AAGACCTTCCATGAGACACTGGATTGTTGTGGAAGCAGCAC 41bp

SS14. CCTGACCGCCCTGACCACCAGCGTGCTCAAGA 32bp

SS15. ACAATCTGTGTCCTAGCGGATCCAACATCATTTCCAACCTGT 42bp

SS16. TCAAAGAGGATTGCCACCAGAAAATTGATGATCTGTTTAGCGGC 44bp

SS17. AAGCTCTACCTCATCGGCATCGCCGCCATCGTC 33bp

SS18. GTGGCTGTCATCATGATTTTCGAGATGATTCTCAGCATGGTCC 43bp

SS19. TCTGCTGCGGAATTAGAAACTCCTCCGTCTACGGCG 36bp

SS20. GAACCGAGACCTCCCAAGTGGCTCCCGCTTGAG 33bp

**^┼^**AS1. CTTCTCGCGGCCGCTCAAGCGGGAGCCA 28bp

AS2. CTTGGGAGGTCTCGGTTCCGCCGTAGACGGAGG 33bp

AS3. AGTTTCTAATTCCGCAGCAGAGGACCATGCTGAGAATCATCT 42bp

AS4. CGAAAATCATGATGACAGCCACGACGATGGCGGCGA 36bp

AS5. TGCCGATGAGGTAGAGCTTGCCGCTAAACAGATCATCAATTTT 43bp

AS6. CTGGTGGCAATCCTCTTTGAACAGGTTGGAAATGATGTTGGAT 43bp

AS7. CCGCTAGGACACAGATTGTTCTTGAGCACGCTGGTG 36bp

AS8. GTCAGGGCGGTCAGGGTGCTGCTTCCACAACAATC 35bp

AS9. CAGTGTCTCATGGAAGGTCTTCACGACAGCTTTAGCGTTG 40bp

AS10. TTGGCATCGTCATCCACCACAGCCTGTTGCAGGG 34bp

AS11. CCTGGTCGTAGAACTGTTTCACGTCCTTAGCAATCTGGTCTTT 43bp

AS12. GTTGACAAATCCCCAAATTCCGGCAGCGACTTCACAAGC 39bp

AS13. GAACAGGATGACGAGGCAGGTAAAGAAGGTTCCGAGGA 38bp

AS14. GACATTGGCTCTCTTGAATAGCTCCGTAGCATCCCAGGAATC 42bp

AS15. CGACAAACATCATCACGGCGCCGACGGCAATCAG 34bp

AS16. GATGTAGATTCCCACATAGAATGTATTAGGAGCGGGCTTGTCTCC 45bp

AS17. GAGTTCCAGGTACAGCAGATTTGTTGTCTGGGGGTCATG 39bp

AS18. TCTGAGCCAGAGAGCCACTCCGAGAATCACGCCTC 35bp

AS19. CGGCGAGCCAGAACACAAAGTTGAACACGAACAGCA 36bp

AS20. GGTACTTAATACACTTTGTGCAGCCCTCCACGCCCATGG 39bp

^*^ SS-Sense Strand

^†^AS-Anti-sense Strand
